# Chronic Cystitis of the Aged

**Published:** 1899-01

**Authors:** I. J. Jones

**Affiliations:** Surgeon Texas Confederate Home


### Chronic Cystitis of the Aged.

BY I. J. JONES, M. D., SURGEON TEXAS CONFEDERATE HOME.

Read at Austin District Medical Society, December 22, 1898.

Probably the more correct title for this paper would be Chronic Cystitis from Urinary Obstruction, as it is this character of disease that will principally be discussed, and with which I have principally had experience.

It has been my fortune to have had a large experience in the treatment of this form of disease, at the Confederate Home, and while I do not expect to advance any new ideas, I hope to present, in tangible form, some practical methods for the care and treatment of these unfortunates that may be helpful hints to the inexperienced.

After an eventful sexual life, the man who has arrived at the age of fifty-five is fortunate indeed if he finds himself still able to project his urine, in a good sized stream, from his body, or is able to retain it during the ordinary hours of sleep. Keen and White state that one-third of all males who have passed fifty-five suffer from enlarged prostate, and in fully one-tenth of them the enlargement is of pathological importance. I have been unable to find any statistics of the frequency of organic urethral stricture, but I have no doubt that it is much more prevalent than is usually suspected, and the two conditions I know are frequently coincident. Classes and conditions of life and morality and society also have much to do with this frequency, I have no doubt; but be this as it may, I am sure that more than one-half of the inmates of the institution which I serve has some obstruction to the urinary flow. In more than forty of the two hundred and forty inmates, this condition is of clinical importance. Of this number, at least two-thirds have more or less chronic cystitis.

When from either of the above causes, as well as from the more infrequent conditions of urinary calculus or cystic tumor, the urine is dammed back in the bladder, it soon undergoes ammoniacal change. It is intensely alkaline in reaction, fetid in smell, and loaded with mucus, triple phosphates and epithelium and other debris, of the disintegrating mucous coat. This change in the urine is a decomposition, and in the mucous debris I have always found multitudes of bacteriæ of several different forms which I have never seen fully classified, though the colon bacillus is frequently found. From my observations, however, I suspect the bacilli of fermentation is the pioneer in the mischief. This urine is so acrid that it erodes the skin with which it comes in contact. Frequently it attacks the mucous membrane of the prepuce, and keeps it in a constant state of inflammation. The urine, usually, constantly dribbles away, and thus, unless measures are taken to prevent it, saturates the surrounding parts, and thus we frequently find the skin covering these parts in a state of intense irritation or inflammation. Of course the mucous coat of the bladder, subjected to the same irritation, has long since become inflammed. As this succession of events progresses, the muscular coat hvpertrophies and contracts irregularly, the mucous coat is swollen and puffy, and the bladder wall is frequently thrown into a number of pockets and sacculi. These pockets are not thoroughly emptied even after thorough catheterization, and their contents are left to further decompose and communicate their decomposition to the fresh urine as fast as it is discharged into the bladder. Sooner or later, by careless use of the catheter or from some foci in the urethra, the pus bacillus reaches this inviting field, and we have an acute inflammation—acute cystitis. If the patient does not succumb to this, it gradually abates and merges into a chronic form of inflammation. During this attack, the inflammation is peculiarly liable to spread to the pelvis and thence along the tubules themselves. When this occurs, death soon follows. One of the accidents most to be dreaded, especially after pus is formed in the bladder, is sudden and complete retention. This may be caused by paralysis of the bladder from over-distension, when it can be easily relieved with the catheter. It may be caused by stranguary, which is a grave occurrence, but in careful hands, the catheter, together with appropriate remedies, such as hot fomentations to the perineum, enemas of starch and laudanum and morphine and atropia, may give relief.

But if a stricture or prostatic obstruction suddenly becomes impassible, there is but one remedy—cystotomy—and if that is to be of any avail it must be done at once. I have only been convinced of this after having lost several patients, more than one of whom I am now convinced could have been saved by timely operation.

With this operation I have but little experience, and that by the supra-pubic route. My patient is still alive. The later authorities seem to prefer the perineal route for this operation, but for what reason I cannot see, as in opening the bladder for any other purpose the tendency is decidedly in favor of the supra-pubic operation. If the patient is not carried away by one of these accidents, he will die by slow exhaustion or intercurrent disease. Now let us see what are the essential links in this pathological chain, that we may apply appropriate and timely relief.

The first link is obstruction and partial retention. For this condition the first thing to consider is, can the condition be removed? If from stricture, we should attempt—very carefully—to dilate with sounds, or if operable, to divide or cut it. Extreme care should always be used with instruments, lest infection occur. Being unable to immediately remove the cause of the obstruction, then catheterism should be begun. This being by far the most important treatment for the disease in all its stages, and being usually in the hands of the patient, requires careful consideration. The first thing is to select the proper kind of instrument. This should be of the largest size that will pass, as there is much less danger in this than from a smaller one, and it is easier to introduce. The best material is soft rubber, if it can be passed, and it can almost always be passed if any can. In obstruction from enlarged prostate, however, a silver instrument with the prostatic curve is most useful. I would, however, never use a metallic instrument if the soft rubber can be passed. Traumatism, more or less, is almost inevitable in passing a metallic instrument through a tough and inelastic stricture, or through the tortuous channel caused by prostatic hypertrophy. By carefully educating your patient in the intelligent, systematic and cleanly use of a proper catheter, you confer upon him—I measure my words—the greatest benefit in your power. My rule as to frequency of drawing the urine is in inverse ratio to the amount of residual urine, i. e., if, after urination, the catheter brings away eight ounces of urine or more, the catheter should be passed once in four hours; if six ounces, once in eight hours; if four ounces, once in twelve hours; if two ounces, once in twenty-four hours. The catheter and parts should be aseptic. The best lubricant is carbolized oil, one in twenty.

The next link is the fermentation and decomposition of the retained urine. For this condition both local and general treatment may be used with benefit. The local treatment consists in washing the bladder with various solutions, the only ones of any benefit in my experience being silver nitrate, two to ten grains to the ounce, according to the patient's susceptibility. Boric acid in saturated solution, hydrogen peroxide, ten to fifty per cent., and ichthyol, ten to twenty-five per cent. I believe the best apparatus for irrigating the bladder is an ordinary fountain syringe and Y. The Y is inserted directly into a soft rubber catheter, or connected with a metallic one by a piece of rubber tubing. This Y has a sliding cut-off, so that when you wish it the water flows without interruption from the syringe to the bladder. When the bladder is filled the cut-off is changed, the discharge pipe lowered until it is below the level of the bladder, when the bladder will be rapidly emptied. This process should be several times repeated at each seance, so that all decomposing detritus shall be thoroughly removed. I object to the tube and funnel, ordinarily recommended in the text-books, first on account of its inconvenience both as to portability and ease, and secondly, it is a dangerous source of infection. The urine flowing backward through the instrument, it is liable to infection, and cannot be cleansed.

Of the solutions mentioned above, the boric acid should be used on all ordinary occasions, the silver solution should be used when it is desired to stimulate the mucous membrane, the peroxide solution when there is much pus, and the ichthyol in all painful conditions. Much can be done by internal medication judiciously employed. The indications to be met are to secure as far as possible a bland aseptic, and nominally-acid condition of the urine,and to stimulate the mucous coat. To render the urine bland, diuretics are used to increase the quantity. In this connection, I desire to call attention to the great and real danger attending the use of two remedies almost universally recommended in our standard works, viz.: alkaline diuretics and turpentine. When, as we have seen, the alkalinity of the urine is one of the prime factors in the causation of the disease, how nonsensical the idea of administering remedies that will increase the alkalinity! This is not fanciful. In 1896, December 28, Mr. M. Duncan, an inmate of the Confederate Home, a sufferer from chronic cystitis, having an attack of indigestion, took several glasses of a solution of bicarbonate of soda. The next morning he had a violent exacerbation of the symptoms of the disease, including great tenesmus and greatly increased irritability and difficulty of urination. This necessitated a more frequent use of the catheter. This resulted, in a few days, in a careless introduction, the bladder was infected, and a violent acute cystitis, resulting in death, followed. Another inmate, Jeptha Williams, suffering with chronic cystitis, took several small doses of turpentine. Stranguary occurred, and before this could be relieved, he died. In my judgment, both remedies are absolutely to be avoided. Buchu, uva ursa, squills, triticum repens, and many other drugs, answer equally well as diuretics, without increasing the alkalinity of the urine. The best of all drugs for the treatment of these conditions is kava-kava. My attention was first called to the pre-eminent value of this drug by the use of a nostrum containing it, by one of my patients. I saw that he was deriving real benefit, and putting him upon the fluid extract of kava-kava, found the benefit to continue. According to Beaumont, Small and Cerna, kava-kava possesses the following therapeutic properties: Nervous stimulant, diuretic, tonic, and locally anæsthetic. It is also strongly resinous, and has therefore the same value as copaiva and turpentine, without their disadvantages, and we also gain the tonic effects of the kava-kava. With this drug we can meet the indications for a diuretic and stimulant of the mucous coat. There are only three remedies generally recommended for the remaining indications, namely, to render the urine acid and antiseptic, boric acid, salol, and benzoic acid. Boric acid interferes with the stomach, and hence the choice lies between the two latter. Benzoic acid, in my observation, is much more effective in rendering the urine acid, and is equally as efficient as an antiseptic. In addition to this, it is a solvent of the phosphates, thus preventing phosphatic deposits and calculi, peculiarly liable to occur in this disease. When these facts became patent to me, I desired to combine the two remedies, but found an insuperable difficulty on account of the resinous character of the kava-kava.

This difficulty has been thoroughly overcome, at my suggestion, by Messrs. Parke, Davis & Co., and they at the same time were able to obscure the bitter nauseous taste of the kava-kava, combining the two into a pleasant, palatable elixir containing seven and one-half grains of the benzoic acid and thirty minims of kava-kava to the fluid drachm. I believe that this can now be obtained, under the name of Elixir Kava-Kava Co. This, in my hands, has proven the most valuable internal treatment.

The dribbling, excoriating urine is one of the most annoying conditions we have to meet. Various mechanical devices in the way of urinals have been devised. In most cases, however, I have found a folded cloth, saturated with five per cent., equally as useful, and not nearly so inconvenient nor offensive.

It will be observed that I have not followed the arrangement nor subject matter of the text-books, and I hope gentlemen who kindly discuss the paper, will discuss the suggestions made, upon their merits, and not by the standard of classical authority.

