# Erratum

**Published:** 1899-01

**Authors:** 


**Erratum.**—In Dr. Jones' article, in third line from bottom on page 359, for "saturated with five per cent.," read "saturated with a five per cent. formaline solution."

